# miRge 2.0 for comprehensive analysis of microRNA sequencing data

**DOI:** 10.1186/s12859-018-2287-y

**Published:** 2018-07-23

**Authors:** Yin Lu, Alexander S. Baras, Marc K. Halushka

**Affiliations:** Department of Pathology, Johns Hopkins University SOM, 720 Rutland Avenue/Ross Bldg. Rm 632B, Baltimore, MD 21205 USA

**Keywords:** miRNA, Small RNA-seq, Alignment, isomiR

## Abstract

**Background:**

miRNAs play important roles in the regulation of gene expression. The rapidly developing field of microRNA sequencing (miRNA-seq; small RNA-seq) needs comprehensive, robust, user-friendly and standardized bioinformatics tools to analyze these large datasets. We present miRge 2.0, in which multiple enhancements were made towards these goals.

**Results:**

miRge 2.0 has become more comprehensive with increased functionality including a novel miRNA detection method, A-to-I editing analysis, integrated standardized GFF3 isomiR reporting, and improved alignment to miRNAs. The novel miRNA detection method uniquely uses both miRNA hairpin sequence structure and composition of isomiRs resulting in higher specificity for potential miRNA identification. Using known miRNA data, our support vector machine (SVM) model predicted miRNAs with an average Matthews correlation coefficient (MCC) of 0.939 over 32 human cell datasets and outperformed miRDeep2 and miRAnalyzer regarding phylogenetic conservation. The A-to-I editing detection strongly correlated with a reference dataset with adjusted R^2^ = 0.96. miRge 2.0 is the most up-to-date aligner with custom libraries to both miRBase v22 and MirGeneDB v2.0 for 6 species: human, mouse, rat, fruit fly, nematode and zebrafish; and has a tool to create custom libraries. For user-friendliness, miRge 2.0 is incorporated into bcbio-nextgen and implementable through Bioconda.

**Conclusions:**

miRge 2.0 is a redesigned, leading miRNA RNA-seq aligner with several improvements and novel utilities. miRge 2.0 is freely available at: https://github.com/mhalushka/miRge.

**Electronic supplementary material:**

The online version of this article (10.1186/s12859-018-2287-y) contains supplementary material, which is available to authorized users.

## Background

MicroRNAs (miRNAs) are short, single-stranded RNAs that post-transcriptionally regulate gene expression via mRNA decay and/or translational repression [1, 2]. MiRNAs are transcribed by RNA polymerases II and III, generating precursors that undergo a series of cleavage events to form mature miRNAs [3]. Around 30 to 60% of all human protein coding genes are regulated by miRNAs [4], involved in almost all biological process ranging from development to metabolism to cancer [5–7].

With the continued popularity of small RNA sequencing to characterize miRNAs, much attention has been focused on miRNA alignment software. In 2015 we introduced miRge, a fast, multiplexing method to align miRNAs and other RNA species to expressed libraries [8]. Since that time, a number of developments in the field have occurred necessitating improvements to this alignment tool.

The number and classification of true miRNAs has become controversial. miRBase, the central resource for miRNA curation, lists 2656 human miRNAs in their recently updated version (v22) [9]. Other manuscripts have listed thousands more putative novel miRNAs [10–12] including new passenger miRNA sequences of known miRNAs. However, the MirGeneDB group has indicated, using strict criteria, that only 586 human miRNA genes (1171 miRNA 5p and 3p strands) exist, calling into question the continued search for novel miRNAs and perhaps the loose methods employed to designate short RNAs as miRNAs from deep RNA-seq data [13].

In recent years, there has also been an increased awareness and value placed on isomiRs. IsomiRs are categorized into three main classes: 5′ isomiRs, 3′ isomiRs and polymorphic isomiRs, with 5′ and 3′ isomiRs subclassified into templated and nontemplated modifications [14]. The 5′ and 3′ isomiRs are the result of imprecise and alternative cleavage during the precursor miRNA (pre-miRNA) processing, post-transcriptional modifications, and/or editing by various post-transcriptional enzymes including exoribonucleases and nucleotidyl transferases [15–19]. IsomiRs are beginning to be considered as more selective than just miRNA expression levels and must become well-characterized [20] and taken into account for alignment strategies [21]. True internal modifications (not technical artifacts) are generally the result of adenosine deaminase (ADAR) acting on RNA to cause an A to I modification [22] as noted in a variety of RNA species. Recently, a call to develop a consistent nomenclature for isomiRs using a. GFF3 file format has been made.

In response to these advancements, we now report major improvements in the 2.0 version of miRge. These include a highly-specific novel miRNA detector based on a machine learning algorithm, a standardized GFF3 isomiR reporting option, and an A-to-I (ADAR1) modification detector. Smaller revisions have been made to the algorithm and libraries to improve miRNA and tRNA calling, increase flexibility of reporting and unification of the code base to Python for ease of programming and allowing for the implementation of miRge 2.0 into the bcbio-nextgen framework. Bcbio-nextgen is a shared-community Python-based toolkit for pipelining and automated analysis of deep sequencing data (https://github.com/bcbio/bcbio-nextgen). We report the improvements and comparisons to other tools below.

## Implementation

### Sequence databases and software dependencies

miRNA libraries were obtained from both miRBase.org [9] and MirGeneDB [13, 23]. mRNA and noncoding libraries were obtained from Ensembl (www.ensembl.org) and other sources (See Additional file 1: Extended Materials and Methods). miRge 2.0 was written in Python (2.7.12) and utilizes a number of tools and libraries including Bowtie (v1.1.1) [24], RNAfold (v2.3.5) [25], SAMtools (v1.5) [26], cutadapt (v1.11) [27], biopython (v1.68), sklearn (v0.18.1), numPy (v1.11.0), SciPy (v0.17.0), pandas (v0.21.0), reportlab (v3.3.0) and forgi (v0.20). (See Additional file 1: Extended Materials and Methods), which are included in an installer. The entire package is available through Bioconda and https://github.com/mhalushka/miRge. miRge 2.0 runs on a Linux platform (Ubuntu 16.04.3).

### miRge 2.0 workflow

**Fig. 1:**
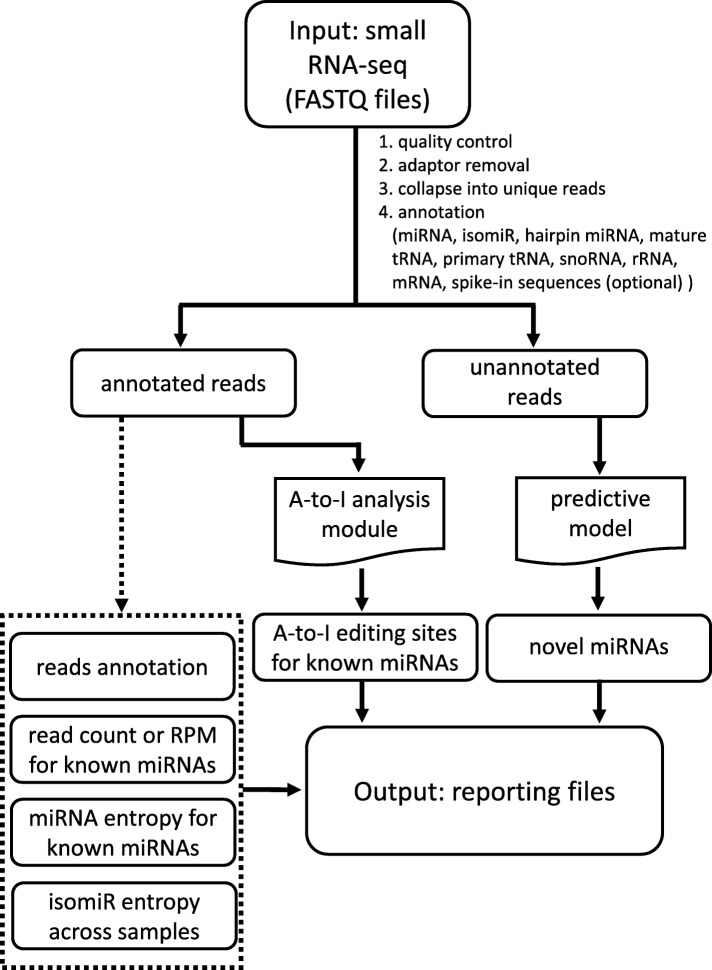
Workflow of miRge 2.0. It illustrates the flow chart from input to output. The models of A-to-I editing sites for known miRNAs and novel miRNAs detection are newly added functions, while the original outputs are shown in dashed box

Figure 1 shows the workflow of miRge 2.0. In Fig. 1, similar to the original miRge, the input FASTQ (or FASTQ.GZ) file(s) undergo prealignment steps of quality control, adaptor removal (cutadapt v1.11) and collapse into unique reads. Their observed counts are subsequently merged across all unique samples [8]. This file is then annotated against multiple search libraries: mature miRNAs, miRNA hairpins, mRNAs, mature & primary tRNAs, snoRNAs, rRNAs, other non-coding RNA, and (optional) known RNA spike-in sequences [28, 29]. A full rationale of the method was given previously [8] and additional modifications are described in “Improvements of miRge 2.0” and in the Additional file 1: Extended Material and Methods.

### Datasets to model novel miRNA detection

Sequencing datasets from 17 tissues in human and mouse (adrenal, bladder, blood, brain prefrontal cortex, colon, epididymis, heart, kidney, liver, lung, pancreas, placenta, retina, skeletal muscle, skin, testes and thyroid) were retrieved from the NCBI Sequence Read Archive (SRA) (Table 1).

**Table 1:** Data sets for constructing the predictive model in human and mouse

Tissue Type	SRA References in human	SRA References in mouse
Adrenal	SRR944031, SRR944034	SRR3653309, SRR3653310
Bladder	SRR333658, SRR333674	SRR3652859, SRR3652860
Blood	SRR837475, SRR837477	SRR5241767, SRR5241768
Brain Prefrontal Cortex	SRR1635903, ERR409900	SRR3540303, SRR3540304
Colon	SRR837839, SRR837842	SRR1973865
Epididymis	SRR384894	NA
Heart	SRR553574, ERR038425	SRR5832818, SRR5832819
Kidney	SRR553575, ERR038420	SRR3652244, SRR3652245
Liver	ERR038413, ERR038410	SRR5832837, SRR5832838
Lung	SRR372648, SRR372650	SRR5059366, SRR5059367
Pancreas	ERR852097, ERR852099	SRR1973869
Placenta	SRR567637, SRR567638	NA
Retina	ERR973611, ERR973613	SRR1427160, SRR1427161
Skeletal Muscle	SRR1635908, SRR1820680	SRR3651659, SRR3651660
Skin	SRR2174513, SRR2174517	SRR3402126, SRR3402132
Testes	SRR333680, SRR553576	SRR1647951, SRR1647953
Thyroid	SRR1291267, SRR1291269	NA

These samples were processed through miRge 2.0 to identify the different RNA species for machine learning controls. MirGeneDB miRNAs were used to assemble positive clusters (known miRNAs). RNAs in the categories of tRNA, snoRNA, rRNA or mRNA were used to assemble negative clusters (known non-miRNAs). Sequences in repeat elements were excluded. The details regarding the final selection of RNA species used are listed in “Generation of read clusters” and in the Additional file 1: Extended Materials and Methods.

### Clustering reads to determine features for model construction and novel miRNA detection

**Fig. 2:**
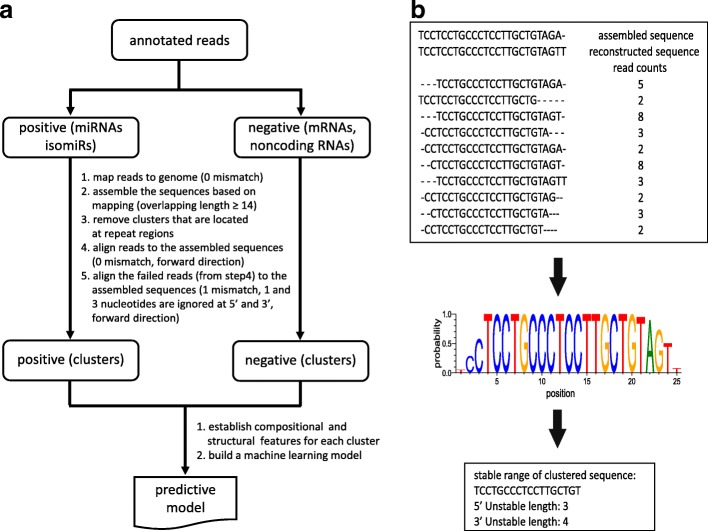
The process of construction of the predictive model. **a** The building of the predictive model composed of data preparation, feature calculation, feature selection and machine learning model training. (Key parameters are in parentheses.) **b** Schematic diagram of generating a stable range of clustered sequences in a cluster. The sequences in the cluster were aligned against the assembled sequence. The probability of the major nucleotide at each position was computed. A threshold of 0.8 was selected to determine the stable range of the cluster sequence

To build a predictive model and for novel miRNA detection, unmapped reads are aligned and clustered to the genome. Figure 2a illustrates this process, specifically during the development of the predictive model, where known miRNAs and known non-miRNAs are processed through multiple steps. During novel miRNA detection, all reads are processed as one, features are determined and these are then fed into the predictive model, rather than being used to build the predictive model. For both development and use of the predictive model, structural features (Additional file 2: Table S1) were generated from the clusters. These methods are described in further detail in the Additional file 1: Extended Materials and Methods.

### Calculating compositional features of read clusters

miRNAs have a characteristic processing pattern by DICER and DROSHA to create a unique, but complicated family of reads and isomiRs. The 5′ ends of the family of reads tends to consistently begin at the same nucleotide, the 3′ end tends to be variable and nucleotide additions of uracils (U/T) or adenines (A) are frequently seen here. Non-miRNAs tend to not share these features, so this difference can be exploited (Additional file 3: Figure S1). In order to codify these patterns, several features of read clusters were defined as follows: 1) the 5′ and 3′ unstable length of the cluster; 2) genome (DNA) nucleotide proportion at the positions − 3, − 2, − 1 of 5′ and + 1, + 2, + 3, + 4, + 5, + 6 of 3′ in the stable range of the cluster sequences; 3) A, T, C, and G percentages in the expressed RNA at the positions − 3, − 2, − 1 of 5′ and + 1, + 2, + 3, + 4, + 5, + 6 of 3′ in the stable range of the cluster sequences. In addition, sequence type count, total read count and the proportion of reads that are an exact match to the cluster sequences were calculated as well.

### Prediction models for novel miRNA detection

We generated measurable features associated with read cluster composition and precursor miRNA structures. These features are listed in Additional file 2: Table S1. The discrimination power of each feature was ranked by its Minimum Redundancy Maximum Relevance (mRMR) score. We applied forward stepwise feature selection [30], to subselect the most informative features.

To test the model for robustness, the dataset was randomly split into training and validation sets at the ratio of 4:1 in 10 replicates. Standardization was performed to scale the features into zero mean and unit variance. The parameters of the estimator were optimized by 10 fold cross-validated grid-search over a parameter grid. The searching space of C and gamma in radial basis function kernel of SVM [31] were {0.0001, 0.001, 0.01, 0.1, 1.0, 10.0, 100.0, 1000}. The SVM model was implemented by scikit-learn Python package (http://scikit-learn.org). Matthews correlation coefficient (MCC) was used to evaluate the performance of the training model. The models were additionally tested on 12 rat samples (Additional file 4: Table S2).

### A-to-I editing analysis

We utilized the mapped output file to identify all reads corresponding to each miRNA for A-to-I editing, as noted as an A to G change. Four exclusion criteria were made to reduce false positive A-to-I identifications based on possible and known sequence similarities and alignment problems. We excluded the putative A-to-I signals if: 1) the locations where similar miRNA families or miRNA SNPs that have A/G differences could be mistaken for A-to-I changes (i.e. nucleotide position 19 in let-7a-5p and let-7c-5p which differ only by an A/G variation and miR- 548al which has a SNP (A-to-G) at position 8 with the frequency of 0.18); 2) the 455 miRNAs found in repeat elements which could give false positives (i.e. miR-6503-3p is located in a MTL1D long terminal repeat.); 3) the miRNAs where the RPM of the canonical sequence is less than 1; 4) the miRNAs where the corresponding one nucleotide switched sequence (A to G) can be aligned to more than 1 location in the genome with trimming the last two nucleotides at 3′. Further description is present in the Additional file 1: Extended Materials and Methods.

### GFF3 isomiR reporting

The increased awareness and interest in isomiRs is challenged by the lack of consistent isomiR reporting. As a result, a consensus standard has been developed by the miRTop consortium utilizing CIGAR values (https://samtools.github.io/hts-specs/SAMv1.pdf). This GFF3 formatted output reports on each isomiR sequence and its relationship to the miRNA precursor.

### Comparison to other novel miRNA tools

Currently, miRDeep2 [32] and miRAnalyzer [33] are two prevailing tools for the prediction of novel miRNAs. In our annotation comparison study, default parameters were utilized except that the ‘-l’ was set to be 17 in the mapper.pl for miRDeep2 and default parameters were utilized in miRAnalyzer. In our prediction comparison study, new FASTQ files were generated from the unmapped read data of an original miRge run. Default parameters were utilized when running miRDeep2 and miRAnalyzer. Two metrics of novel miRNAs were used to compare three tools: PhyloP score and quality score. Basewise conservation scores across miRNAs were calculated from PhyloP data downloaded from http://hgdownload.cse.ucsc.edu/goldenPath/hg38/phyloP20way/ [34] using the PHAST package [35]. For each miRNA, the mean of PhyloP values across its length was calculated. The quality scores for each miRNA by each tool was defined by: 1 - (ranking percentile by the tool).

### Hardware

All processing was performed on a workstation with 56 CPUs (dual Intel(R) Xeon(R) E5–2690 v4 at 2.60GHz) and 256GB DDR4-RAM. Novel miRNA modelling was performed using 32 CPUs. For speed testing, the number of CPUs in running original miRge, miRge 2.0, miRDeep2 and miRAnalyzer were 5, 5, 5 and 1, respectively. Due to a java incompatibility on the workstation, miRAnalyzer was run on a desktop with 4 CPUs (Intel(R) Core(TM) i7–6700 CPU at 3.40GHz) and 16GB DDR4-RAM.

## Results

### Improvements of miRge 2.0

The major improvements of miRge 2.0 consist of a novel miRNA detection method, improved alignment parameters, and the reporting of A-to-I changes in the sequence. These are described below, while smaller improvements are reported here. Utilizing updated search parameters, miRge 2.0 is able to annotate reads more precisely. In human data, using the miRBase v22 library, miRge 2.0 will align to 2817 miRNAs of which 149 are merged due to a similarity of their sequences. Although most miRNA alignment tools are agnostic to exact (canonical) or mismatched alignments (nontemplated isomiRs), miRge 2.0 sets a threshold (default value: 0.1; range 0–0.5) of the proportion of canonical reads to all reads for any given miRNAs. This can eliminate over reporting of miRNAs in which too high a percentage of sequences are nontemplated isomiRs, likely from other genomic loci or species contamination. miRge 2.0 also provides an optional GFF3 file report, which implements the miRTop guidelines for isomiR reporting utilizing CIGAR values. These can be used for isomiR-driven analyses. Additionally, the GFF3 data file is easily incorporated into other analysis pathway software including the bcbio-nextgen framework. miRge 2.0 also generates a .csv and .pdf file report of summary statistics; replacing a html report which was more difficult to process for tabular information. We also made several revisions to the search libraries. For the miRBase-based alignment search, we included additional SNP information in the miRNA library based on the updated miRNASNP database [36]. For the miRBase based searches, we have also included 161 5p or 3p miRNAs that are the complement of known miRBase miRNAs for which the passenger strand was detected recently [11]. Thus we have expanded our miRBase search library from 2656 miRNAs in our original method to 2817 miRNAs currently. We have also built a MirGeneDB 2.0-based alignment library that is corrected for SNP information for those investigators seeking this more specific set of miRNAs. We have also improved the tRNA alignments by adding “CCA” to the 3′-end of mature tRNA sequences and the precursor tRNA sequences at the 3′-end. For any alignment, we have added an optional spike-in RNA library search based on two popular sources of spike-in normalization [28, 29]. This search can easily be expanded to capture newer spike-in normalization methods as they appear. All options to call in miRge 2.0 are shown in Additional file 5: Table S3.

### Speed and annotation comparison of original miRge and miRge 2.0

We performed tests of speed and annotation function of miRge 2.0 using six datasets. Both miRBase and MirGeneDB based libraries were analyzed although novel miRNA detection and A-to-I analysis were not performed. We found the processing time of miRge 2.0 was similar to the original miRge although bowtie searching libraries and searching parameters were adjusted (Table 2).

**Table 2:** Annotation comparison of the first version of miRge, miRge 2.0, miRDeep2 and miRAnalyzer

Tissue/Cell	SRA References	Alignment Tool	Processing time	miRNA Reads	Unique miRNAs	miRNAs > 10 RPM
Human Adipose Tissue	SRR772563	miRge - mb	35 s	2,041,433	484	240
		miRge 2.0 - mb	36 s	2,039,835	477	238
		miRge 2.0 - MDB	35 s	2,034,710	390	220
		miRDeep2	9.3 min	1,981,793	598	224
		miRAnalyzer	30 s	1,752,855	689	243
Human Alpha Cell	SRR1028924	miRge - mb	14.6 min	44,124,580	920	293
		miRge 2.0 - mb	15.6 min	43,880,855	911	279
		miRge 2.0 - MDB	15.0 min	43,752,598	583	261
		miRDeep2	56.0 min	42,326,135	864	267
		miRAnalyzer	18.4 min	34,349,816	1124	281
Human Beta Cell	SRR873410	miRge - mb	6.5 min	26,196,298	896	297
		miRge 2.0 - mb	6.6 min	26,197,845	889	291
		miRge 2.0 - MDB	6.5 min	26,130,904	585	274
		miRDeep2	34.1 min	23,280,604	754	273
		miRAnalyzer	8.0 min	14,240,669	1113	289
Mouse Stomach Tissue	SRR3653378	miRge - mb	2.0 min	7,063,128	804	457
		miRge 2.0 - mb	2.3 min	7,175,534	806	420
		miRge 2.0 - MDB	2.2 min	7,094,217	578	378
		miRDeep2	18.5 min	6,738,987	748	387
		miRAnalyzer	2.5 min	6,818,220	1086	423
Mouse Epididymal Epithelial Cell	SRR2075702	miRge - mb	3.0 min	1,394,193	435	364
		miRge 2.0 - mb	3.6 min	1,387,591	411	290
		miRge 2.0 - MDB	3.4 min	1,381,670	360	271
		miRDeep2	24.4 min	1,367,627	402	212
		miRAnalyzer	3.0 min	925,019	532	270
Mouse B3 Cell	SRR2960463	miRge - mb	3.7 min	9,515,760	604	322
		miRge 2.0 - mb	3.9 min	9,612,571	606	282
		miRge 2.0 - MDB	3.9 min	9,553,713	359	227
		miRDeep2	32.6 min	8,321,228	487	251
		miRAnalyzer	4.2 min	6,856,264	819	289

Key: mb = miRBase; MDB = MirGeneDB. Starting read counts: SRR772563 = 2,373,604 reads; SRR1028924 = 82,497,527 reads; SRR873410 = 33,233,648 reads; SRR3653378 = 9,587,887 reads; SRR2075702 = 13,890,643 reads; SRR2960463 = 17,652,076 reads

The number of detected miRNAs was slightly decreased as well. The alignment speed was essentially the same as miRAnalyzer and significantly faster than miRDeep2. The discovery of novel miRNAs is more time and memory intensive, as expected. For the dataset SRR553572 with 25.7 million reads, to identify novel miRNAs, the calculation time and maximum memory consumption were 17 mins and 6.7 GB RAM respectively.

### A-to-I editing analysis

**Fig. 3:**
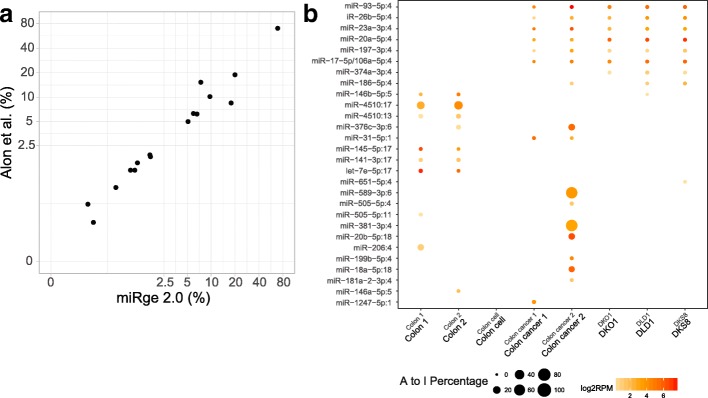
A-to-I analysis. **a** The A-to-I proportion of the sites is strongly correlated with a reference dataset analysis with adjusted R^2^ of 0.96 in the log-log plot. **b** The output of miRge 2.0 showing an illustrated heat map of miRNA A-to-I editing sites across colon tissue, primary colon cell, colon cancer tissue and colon cancer cells from multiple sources

To evaluate the accuracy of A-to-I editing analysis, we performed A-to-I analysis using a pooled human brain sample (SRR095854) and compared the results to prior published data on this sample [37]. We identified 19 significant A-to-I modification sites compared to 16 reported in the reference paper. Comparing the two sets of results, the adjusted R^2^ of A-to-I proportion of these shared 16 sites was 0.96 and the slope of the linear regression was 0.99 indicating high reproducibility between our method and the established method (Fig. 3a). We then performed a new A-to-I editing analysis across colon tissue (Sequence Read Archive samples: SRR837842 and SRR837839), colon epithelial cells (SRR5127219), colon cancer (SRR1646473 and SRR1646493), and the colon cancer cell lines DKO1 (SRR1917324), DLD1 (SRR1917336) and DKS8 (SRR1917329). Significant miRNA editing sites with A-to-I percentage ≥ 1% in at least one sample were shown in Fig. 3b, with the data indicating differences between tumor and normal cells in ADAR activity [38].

### Validation of the predictive model

**Fig. 4:**
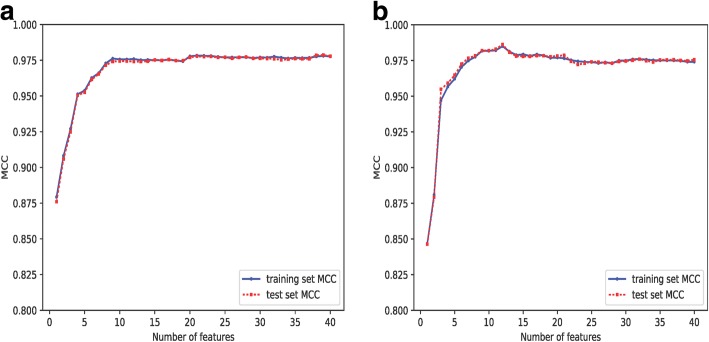
Model performance on top 40 features for training and validation sets for human **a** and mouse **b** miRNA discovery. Each dot stands for the mean value of Matthews correlation coefficient (MCC)

To determine the optimal number of features to use in the human and mouse predictive model, the MCC for the training and validation sets for the top 40 ranked features based on mRMR scores are shown in Fig. 4. For human data, when the number of features reached 21, the mean value of MCC of training and validation set approached the maximum and became stable. These top features are listed in Table 3.

**Table 3:** Top 21 features in human predictive model. Hairpin structural features are labeled in italics, while read compositional features are not

Rank	Feature name	Description of the feature
1	*count_bindings_in_miRNA*	Number of bindings in the stable range of sequences
2	exactMatchRatio	The proportion of reads that are an exact match to the cluster sequence in the cluster
3	*pair_state_No*	Whether there is another stable range of sequences located at the other arm of precursor
4	*mFE*	Minimum free energy (MFE) of the precursor
5	head_minus3_TemplateNucleotide_percentage	Proportion of genomic templated nucleotide at position −3 relative to the 5′ end of the stable range of the cluster sequences
6	*hairpin_count*	Number of hairpin loops in the precursor
7	*stem_length*	Stem length of the precursor
8	*distanceToloop*	Distance between the stable range of sequences and the terminal loop
9	*percentage_PairedInMiRNA*	Number of bindings in the stable range of sequences divided by its length
10	headUnstableLength	5′ unstable length of the cluster
11	*pair_state_Yes*	Whether there is another stable range of sequences located at the other arm of precursor
12	tail_plus2_A_percentage	Proportion of non-templated adenine (A) at position + 2 relative to the 3′ end of the stable range of the cluster sequences
13	head_minus2_TemplateNucleotide_percentage	Proportion of genomic templated nucleotide at position −2 relative to the 5′ end of the stable range of the cluster sequences
14	*binding_count*	Number of bindings in the precursor hairpin
15	tail_plus1_A_percentage	Proportion of non-templated adenine (A) at position + 1 relative to the 3′ end of the stable range of the cluster sequences
16	*armType_loop*	Whether the stable range of sequences is located at the terminal loop if the precursor
17	tail_plus3_A_percentage	Proportion of non-templated adenine (A) at position + 3 relative to the 3′ end of the stable range of the cluster sequences
18	tail_plus5_TemplateNucleotide_percentage	Proportion of genomic templated nucleotide at position + 5 relative to the 3′ end of the stable range of the cluster sequences
19	tail_plus1_TemplateNucleotide_percentage	Proportion of genomic templated nucleotide at position + 1 relative to the 3′ end of the stable range of the cluster sequences
20	*interiorLoopCount*	Number of interior loops in the precursor
21	head_minus1_TemplateNucleotide_percentage	Proportion of genomic templated nucleotide at position −1 relative to the 5′ end of the stable range of the cluster sequences

Among them, there are 11 precursor miRNA structural features and 10 compositional features. The ultimate model was constructed using these selected features. We used 32 human cell data sets to test the model. The positive and negative miRNAs were generated through the same process described above. The predictive result is shown in Table 4. The mean of MCC is 0.94, indicating that the performance of the model in the test set is good.

**Table 4:** Predictive results of 32 human cell data in a test set by the human model

Cell Type	SRA References	AUC	Precision	Recall	MCC
Fibroblast Aorta Adventitia	SRR5127206	0.995	0.983	0.963	0.945
Smooth Muscle Cell Aorta	SRR5127217	0.994	0.981	0.961	0.938
Astrocyte	SRR5127214	0.994	0.98	0.968	0.949
Smooth Muscle Cell Bladder	SRR5127215	0.992	0.971	0.963	0.936
Fibroblast Dermal (Adult)	SRR5127205	0.995	0.983	0.974	0.95
Fibroblast Dermal (Neonatal)	SRR5127225	0.995	0.989	0.96	0.942
Epithelium Keratinocyte (Adult)	SRR5127203	0.994	0.977	0.962	0.934
Epithelium Keratinocyte (Neonatal)	SRR5127208	0.993	0.975	0.942	0.923
Endothelial Aortic	SRR5139121	0.988	0.975	0.932	0.915
Endothelial Umbilical vein	SRR5127213	0.993	0.981	0.954	0.926
Epithelium Bronchial	SRR5127216	0.988	0.974	0.951	0.935
Chondrocyte	SRR5127229	0.995	0.985	0.959	0.944
Endothelial Microvascular	SRR5127201	0.991	0.973	0.957	0.945
Fibroblast Cardiac	SRR5127236	0.992	0.983	0.945	0.94
Melanocyte	SRR5127207	0.995	0.99	0.981	0.954
Epithelium Mammary	SRR5127224	0.99	0.976	0.941	0.927
Epithelium Prostate	SRR5127212	0.992	0.975	0.961	0.948
Epithelium Renal Cortex	SRR5127204	0.988	0.966	0.948	0.927
Epithelium Renal Proximal	SRR5127230	0.992	0.978	0.949	0.936
Stromal cell Prostate	SRR5127226	0.991	0.976	0.963	0.94
Myoblast Skeletal Muscle	SRR5127218	0.99	0.974	0.956	0.932
Epithelium Intestinal	SRR5127223	0.994	0.985	0.973	0.957
Myofibroblast	SRR5127220	0.991	0.987	0.965	0.95
Smooth Muscle Cell Prostate	SRR5127222	0.991	0.978	0.961	0.943
Neuron Dopaminergic	SRR5127234	0.982	0.963	0.922	0.916
Neuron Cortical	SRR5127209	0.986	0.968	0.917	0.917
Mesangial	SRR5127221	0.996	0.986	0.971	0.948
Osteoblast	SRR5127233	0.997	0.986	0.955	0.946
Fibroblast Periodontal ligament	SRR5127227	0.994	0.986	0.962	0.946
Epithelium Renal	SRR5127235	0.992	0.989	0.954	0.936
Epithelium Retinal Pigment	SRR5127210	0.994	0.988	0.974	0.959
Skeletal Muscle Cell	SRR5127202	0.995	0.984	0.96	0.936
Mean		0.992	0.98	0.956	0.939
Std dev		0.003	0.007	0.014	0.012

Meanwhile, in the mouse predictive model, the optimal number of features are 12 which is shown in Additional file 6: Table S4. These 12 features are a subset of the 21 human features used. The performance of a mouse model towards 19 mouse cell datasets are shown in Additional file 7: Table S5 where the mean of MCC is 0.93, indicating that the mouse model performed well on the test dataset.

### Comparison with other novel miRNA detection tools

**Fig. 5:**
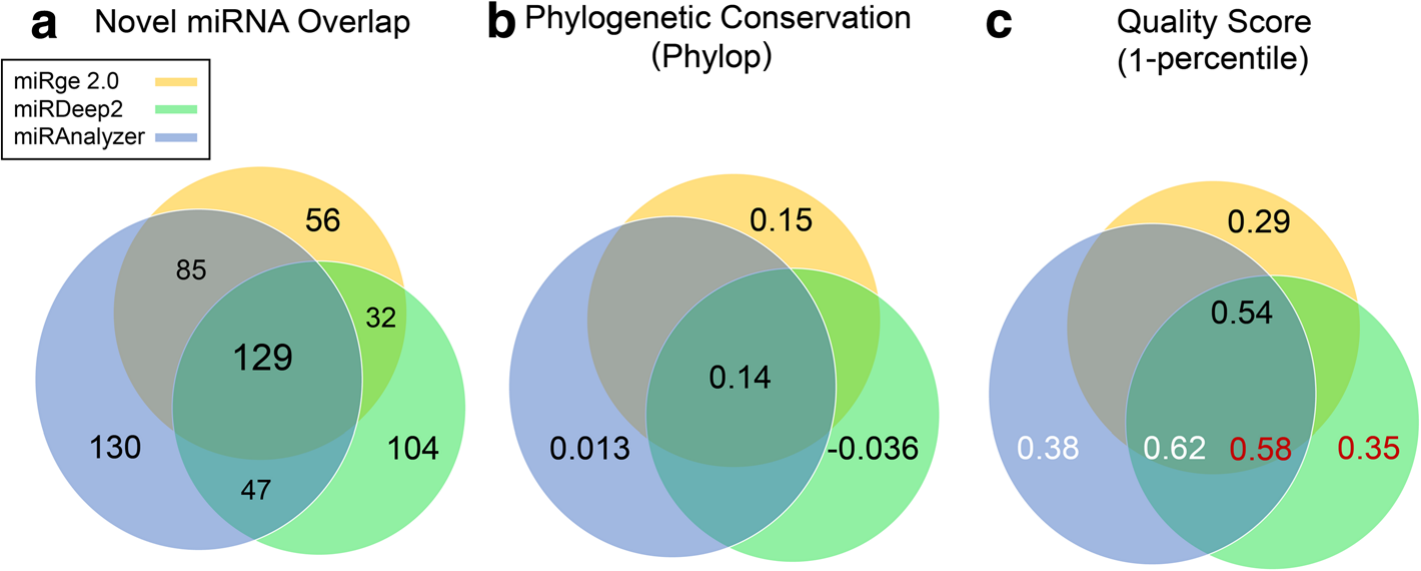
Venn diagram for novel miRNAs predicted by miRge 2.0, miRDeep2, and miRAnalyzer. **a** Overlapped novel miRNAs among the three tools. **b** The average basewise conservation scores across novel miRNAs. **c** The average Quality score across novel miRNAs among the three tools

Using miRge 2.0, we identified 302 RNA species that are putative novel miRNAs from 32 cell types [11]. Referring to these sequences as novel miRNAs, without further validation, may be incorrect terminology. However, without other terminology for these small “true miRNAs” or “miRNA-like RNA species,” we will refer to them as putative novel miRNAs. We then used the same unmapped reads generated from miRge 2.0 as input for miRDeep2 and miRAnalyzer. They predicted 1975 and 18,168 putative novel miRNAs respectively. After thresholding the data from those two tools to the same parameters as miRge 2.0 (≥10 total reads, ≥3 sequences, etc.), there were 312 and 391 putative novel miRNAs remaining. As shown in Fig. 5a, a Venn diagram depicts the overlap among miRge 2.0, miRDeep2 and miRAnalyzer, showing 129 novel miRNAs shared between the three methods. We then calculated the mean PhyloP scores as a measure of nucleotide conservation across primates for the novel miRNAs (Fig. 5b). More conservation was noted for the shared novel miRNAs (0.14) compared to miRAnalyzer (0.013) and miRDeep2 (− 0.036). Conservation was equivocal between the shared novel miRNAs and the miRge 2.0 novel miRNAs (0.15) As all three tools give a quality score to each novel prediction, we compared these values for miRNAs found shared vs. those unique to each method. As shown in Fig. 5c, the overlapped miRNAs ranked higher in quality for each method, further suggesting these 129 are the optimal putative novel miRNAs from the group. The full list of putative novel miRNAs generated by the three tools are available in Additional file 8: Table S6.

### Comparison between the human model and mouse model

We used our SVM model to create an optimal novel miRNA tool for both human and mouse. We also trained a model using combined human and mouse data. We questioned how well those tools could predict novel miRNAs in another mammalian species. We utilized these three (human-only, mouse-only and combined) models on the 12 rat miRNA samples shown in Additional file 4: Table S2. Using known miRNAs and known non-miRNAs, we found the average MCC for the rat samples to be essentially equivalent among the three models (0.95–0.96). The average MCC for testing the human model on the mouse data and mouse model on the human data are 0.93 and 0.89, respectively. Therefore, although same-species modeling might be ideal, the combined human and mouse SVM model is incorporated to be used for other mammalian species in miRge 2.0.

## Discussion

In light of the positive and negative feedback we received for our original miRge tool, we generated an improved 2.0 version. miRge 2.0 has a more robust search, better overall output reporting, more run options, and new parameters for novel miRNA detection and A-to-I editing detection. It can be installed by bioconda and implemented within the bcbio-nextgen framework to better integrate with other software tools. It still remains one of the fastest options for alignment and can multiplex multiple samples in a single run. The new novel miRNA detection tool has reasonable requirements for RAM and can be used widely.

Our data suggests the miRge 2.0 novel miRNA detection tool is more robust than the earlier tools miRDeep2 and miRAnalyzer. As much recent literature suggests, the novel miRNA detection tools have been too open in their parameters, allowing an explosion of novel miRNA reporting, that is likely inaccurate. For novel miRNA detection in established species, less is likely more. We believe that our more strict requirements and unique use of compositional features has improved miRNA discovery and is a better approach going forward. We caution though, that these are putative novel miRNAs and should not be thought of as bona fide miRNAs unless they meet additional parameters [13]. We are also wondering if a novel detection tool built for one mammalian species could be used to detect putative novel miRNAs in other species. Our human and mouse models assayed with the rat data indicates, that, indeed, at least among mammalia, our tool is robust.

We have also tried to make miRge 2.0 more robust to current concerns of the community. Many authors have argued that miRBase—the online repository for miRNAs —is riddled with false positive miRNAs [39–42]. Therefore, we have built a MirGeneDB 2.0-based alignment library, incorporating SNPs, for six species to cater to those investigators seeking a better-defined set of miRNAs. We have reported concerns with using reads per million miRNA reads (RPM) as a normalization tool [43]. Therefore, we have added an optional spike-in RNA library search step for spike-in normalization. Spike-in for miRNA RNA-seq is still in its infancy, so this step can easily be expanded/modified to account for newer spike-in normalization methods. Currently, the sequence libraries of human, mouse, rat, nematode, fruitfly and zebrafish datasets are provided, but miRge 2.0 can be used by individual users to investigate any species by constructing the sequence libraries to incorporate in the miRge 2.0 workflow using our miRge_bowtie_build.py tool.

In our original miRge tool, we accepted that reads could randomly align to highly similar miRNAs, e.g. miR-192-5p and miR-215-5p; thus we reported those together as miR-215-5p/192-5p reads. The cross-mapping of sequencing reads can create false alignments that may be interpreted as sequence or expression alterations which can occur in other alignment tools, as other tools have generally not hand-curated their alignment libraries. Our improvements in miRge 2.0 optimize the number of miRNAs that are clustered together to reduce these random alignment challenges.

With the interest in ADAR activity and A-to-I changes in RNAs, we have added a feature to miRge 2.0 to capture this information. miRge 2.0 performs robustly in identifying these ADAR sites, comparable to other stand-alone programs.

## Conclusion

In summary, miRge 2.0 is an update of our original miRNA alignment tool that more comprehensively and more robustly analyzes miRNA sequencing data. We believe the improvements in miRge 2.0 will be useful to a wide range of scientists who are interested in interpreting small RNA-seq data for miRNA expression patterns.

## Availability and requirements

**Project name:** miRge 2.0.

**Project home page**: https://github.com/mhalushka/miRge

**Operating System:** Linux.

**Programming Language:** Python.

**Other Requirements:** Bowtie (v1.1.1), RNAfold (v2.3.5), SAMtools (v1.5), cutadapt (v1.11), biopython (v1.68), sklearn (v0.18.1), numPy (v1.11.0), SciPy (v0.17.0), pandas (v0.21.0), reportlab (v3.3.0) and forgi (v0.20).

**License:** GNU GPL 3.0.

**Any restrictions to use by non-academics:** none.

## Additional files

Additional file 1: Extended materials and methods on the development of miRge 2.0. (PDF 132 kb)

Additional file 2: **Table S1.** Features calculated for building the novel miRNA detection model. (PDF 46 kb)

Additional file 3: **Figure S1.** Distribution of non-templated nucleotides as a percentage of all 4 nucleotides at both 5′ and 3′ positions relative to the mature miRNA or equivalent non-miRNA sequence (PDF 876 kb)

Additional file 4: **Table S2.** 12 rat samples for evaluating the human and mouse novel miRNA predictive models. (PDF 19 kb)

Additional file 5: **Table S3.** All options to call in miRge 2.0. (PDF 55 kb)

Additional file 6: **Table S4.** Top 12 features in the mouse novel miRNA predictive model. (PDF 7 kb)

Additional file 7: **Table S5.** Predictive results for 19 mouse RNA-seq datasets for novel miRNA detection. (PDF 20 kb)

Additional file 8: **Table S6.** Novel miRNAs detected by miRge 2.0, miRDeep2 and miRAnalyzer. (XLSX 167 kb)
