# Supplementary material for: miRge 2.0 for comprehensive analysis of microRNA sequencing data
Source: BMC Bioinformatics. 2018 Jul 23;19:275. doi: 10.1186/s12859-018-2287-y (PMC6112139; doi:10.1186/s12859-018-2287-y)
Supplement: Supplementary file 1 — Extended materials and methods on the development of miRge 2.0. (PDF 132 kb) [file 12859_2018_2287_MOESM1_ESM.pdf]

Extended Materials and Methods

Sequence databases and software dependencies

miRNA libraries were obtained from both miRBase.org [1] and MirGeneDB [2]. mRNA and noncoding libraries were obtained from Ensembl (www.ensembl.org) unless otherwise noted. Human tRNAs were obtained from the Genomic tRNA Database [3]. Human snoRNA was obtained from the snoRNABase (www-snorna.biotoul.fr). Repeat element locations were obtained from the UCSC genome browser track on Hg38. The redundant sequences in non-miRNA libraries were removed and the regions in these sequences which were identical to mature miRNAs were substituted with Ns. miRge 2.0 was written in Python (2.7.12) and utilizes the following tools and libraries: Bowtie (v1.1.1) [4], RNAfold (v2.3.5) [5], SAMtools (v1.5) [6], cutadapt (v1.11) [7], biopython (v1.68; http://biopython.org), sklearn (v0.18.1; http://scikit-learn.org), numPy (v1.11.0; http://www.numpy.org), SciPy (v0.17.0; https://www.scipy.org), pandas (v0.21.0; http://pandas.pydata.org), reportlab (v3.3.0; http://www.reportlab.com) and forgi (v0.20; https://viennarna.github.io/forgi). An installer incorporating all of these tools except Bowtie, SAMtools and RNAfold is included and the entire package is available through Bioconda. miRge 2.0 runs on a Linux platform (Ubuntu 16.04.3).

miRge 2.0 Workflow

Figure 1 shows the workflow of miRge 2.0. In Figure 1, similar to the original miRge, the input FASTQ or FASTQ.gz file(s) undergo prealignment steps of quality control, adaptor removal (cutadapt v1.11) and collapse into unique reads and their observed counts with subsequent merging across all unique samples [8]. This file is then annotated against these search libraries: mature miRNA, miRNA hairpin, mRNA, mature & primary tRNA, snoRNA, rRNA, other non-coding

RNA, and (optional) known RNA spike-in sequences [9, 10]. A full rationale of the method was given previously [8]. Briefly, the initial alignment to mature miRNAs allows 0 mismatches. Alignments to all other species allows 1 mismatch. In the second alignment to the mature miRNAs (the isomiR step), the Bowtie search was modified from “bowtie -l 15 -5 1 -3 2 -n 2 –f” to “bowtie -5 1 -3 2 –v 2 –f –norc –best –S.” As an update from the original miRge approach, only forward strand direction matching was allowed in the Bowtie step to search miRNAs with greater accuracy [11].

We addressed the effect of reads cross-mapping to more than one miRNA. We approach this by clustering the reads of the two or more similar miRNAs together (ex. hsa-miR-215-5p/192-5p). We made several improvements over the original miRge approach including systematically analyzing sequence similarity and merging miRNAs together if no mismatch is present in the main region of the miRNA. This was hand-curated and experimentally validated by repeated Bowtie alignments investigating random placement of reads. More specifically, Bowtie can be run so that if it aligns a read equally to two different sequences it randomly picks one assignment. Since we pre-cluster identical reads from the FASTQ file, we have essentially forced reads to all be assigned to one sequence over the other. Because of the randomness factor, if one repeats Bowtie alignments, these “equal alignment” reads can be noted to “jump” from one sequence to another. We identified these reads and used that information to cluster similar miRNAs together.

Two new optional modules added in miRge 2.0 are the identification of ADAR A-to-I editing positions in the miRNAs and the search for putative novel miRNAs from unannotated reads (described below). Output files contain: 1) a .csv file containing all the annotated sequences; 2) two .csv files containing reads counts or reads per million (RPM) per miRNA; 3) an optional .csv file containing miRNA entropy and % canonical reads per miRNA; 4) an optional .csv file on the

entropy of each isomiR across samples; 5) a .pdf report file containing an annotation log of the unique sequences identified across the entirety of the sample set analyzed along with per sample information on total reads, sequence length histograms, and the composition of the sample with respect to miRNA, mRNA, ncRNA, genomic, and unaligned reads; 6) an optional .gff file on the miRNAs and isomiRs (including CIGAR annotation) across samples; 7) an optional .csv file containing the identified significant A-to-I editing site in miRNAs and their proportion and adjusted p value; 8) an optional .pdf file showing a heat map of the A-to-I editing sites across samples; 9) an optional .csv report file of each sample containing the identified novel miRNAs; 10) Multiple .pdf files containing the structure of precursor miRNAs, the location, and reads alignment of novel miRNAs.

Datasets to model novel miRNA detection

Sequencing datasets from 17 tissues in human and mouse (adrenal, bladder, blood, brain prefrontal cortex, colon, epididymis, heart, kidney, liver, lung, pancreas, placenta, retina, skeletal muscle, skin, testes and thyroid) were retrieved from Sequence Read Archive (Table 1). These samples were processed through miRge 2.0 to identify the different RNA species for machine learning controls. MirGeneDB miRNAs were used to assemble positive clusters (known miRNAs). RNAs in the categories of tRNA, snoRNA, rRNA or mRNA were used to assemble negative clusters (known non-miRNAs). Sequences in repeat elements were excluded. The details regarding the final selection of RNA species used are listed in “Generation of read clusters.” The collected miRNAs were further subselected by removing those that had less than 3 unique sequences, less than 10 overall reads, and are unable to form putative pre-miRNA structures. This yielded 12,048 and 7,795 known miRNAs (positive clusters) and 52,395 and 7,044 non-miRNAs (negative clusters) for the human and mouse datasets, respectively. To balance the positive and negative

cluster data, 12,048 non-miRNA elements were randomly sampled from the original 52,395 in the human dataset and 7,044 miRNA elements were randomly sampled from the original 7,795 in the mouse dataset.

Clustering reads to determine features for model construction and novel miRNA detection

Figure 2A illustrates the process of construction of the predictive model. The goal of this step is two-fold. The first is to use known positive (miRNA) and negative (non-miRNA) sequences to cluster reads together to a genomic location and allow compositional and structural features to be determined for each cluster. These are used to build a predictive model. Secondly, this method is applied to the unmapped.csv output of user inputted FASTQ data. Clusters from the unmapped reads are used to establish features evaluated by the predictive model to detect novel miRNAs. The steps of this method (as described for developing the model) are: 1) Annotated reads previously from a FASTQ file are classified into positive reads (miRNAs and isomiRs based on miRGeneDB) and negative reads (mRNAs and noncoding RNAs); 2) These raw sequence reads are mapped to the human genome using Bowtie with 0 mismatches, seed length of 25 bp and alignment to 3 or fewer loci and then assembled based on coordinates with perfect alignment. A new sequence cluster is generated based on their overlapping coordinates. To form a cluster, two or more overlapping reads must have the same strand directionality with a minimum overlapped length of 14 bp. We removed assembled cluster sequences with length > 30 bp, a 6+ bp poly-A at the 3’ end, a 6+ bp poly-T at the 5’ end, or if they were located in a repetitive element region; 3) All the reads were then mapped to these assembled cluster sequences with 0 mismatches, seed length of 25, and forward direction. The reads that did not align in this first step were mapped to the clusters in a less stringent manner, in which the first and last 3 nucleotides were ignored, up to 1 misaligned base pairs was allowed, the seed length was set to 15 bp. The most stable region of

each cluster is extracted as a putative mature miRNA in the three steps, shown in Figure 2B. First, all reads were aligned to the cluster; then we calculated the ratio of the read counts of each base along the cluster to the total read number for the cluster and finally set the start and end position of the putative miRNA as the first and last base with a ratio >0.8 of base position reads to total reads of the cluster. 4) We used these read structures to determine the optimal candidate pre-miRNA hairpins based on folding energy (RNAfold) of the surrounding sequence. 5) The compositional features and the structural features of the pre-miRNAs were computed for each cluster. 6) A support vector machine (SVM) model, described below, was built to calculate the probability that a given candidate was a miRNA. 7) The probability of each putative mature miRNA is compared to the known positive or negative miRNA status of the read cluster to develop test statistics.

Generating precursor miRNA structural features for candidate novel miRNAs

From the clustering process described above, genomic positions of the clusters (of known miRNAs, known non-miRNAs, and unmapped reads) were obtained. The precursor (hairpin) candidate structures were generated as follows:

1) If a mapped cluster had no adjacent clusters, we determined a most-likely precursor (hairpin) structure. To do this, we generated two potential hairpin structures. We either added 20 nt upstream and 70 nt downstream or 70 nt upstream and 20 nt downstream of genomic sequence. Then, the secondary structure of the precursor was predicted by RNAfold. Any sequence without a hairpin secondary structure was removed at this step. After prediction, 5 nt was removed from the 20 nt side, and a matching length of sequence was removed from the 70 nt side, such that the hairpin had no overhang. We determined a 15 nt extended length is optimal for determining minimum free energy.

2) Precursor miRNA hairpin structures were discarded, if: a) a read cluster overlaps with the loop by more than 5 bp in the 5p-arm (no overlap is allowed on 3p-arm); b) the pruned pre-miRNA has no hairpin; c) the hairpin has less than 15 bindings in the total precursor structure; d) < 60% of nucleotides in the putative mature miRNA cluster are paired. If both precursor options remain, we chose the precursor with the lowest minimum free energy.

3) For those sequence clusters that had additional nearby clusters (within 44 bp), which could represent 5p and 3p arms, we approached these slightly differently. For these sequences, we assigned the neighbor state of each cluster sequence. To do this, we assigned the distance from the adjoining upstream sequence "seq1" to the target sequence "seq2" as D_1. If there were three nearby sequence clusters, then we determined the distance from the adjoining downstream sequence "seq3" to the target sequence "seq2" as D_2. If $9 <= D_1 <= 44$ and the direction of "seq1" is equal to the direction of "seq2" or $9 <= D_2 <= 44$ and the direction of "seq2" is equal to the direction of "seq3." From each plausible scenario, we generated a precursor structure as described in 1) above and determined the optimal precursor based on the rules of 2) above.

A-to-I editing analysis

We utilized the mapped output file to identify all reads corresponding to each miRNA for A-to-I editing, as noted as an A to G change. First, the reads were aligned against the genome with the last two nucleotides at the 3' end trimmed and allowance of up to one mismatch. Here, we demanded unique best hits (i.e. a read that cannot be aligned to other locations in the genome with the same number of mismatches). Then, for the retained reads that belong to one miRNA and its isomiR, all nucleotide positions in the canonical miRNA, except the terminal 5 bp were screened for A to G changes based on a binomial test considering the expected sequencing error rate (0.1%), as described [12]. A Benjamini-Hochberg-corrected P-value [13] was calculated for each site on

the miRNA. The A-to-I editing level was defined as the proportion of the mapped reads containing the edited nucleotide relative to the total mapped reads at the given location. Finally, we excluded the putative A-to-I signals based on four criteria described in the main manuscript.

Extended Materials and Methods Citations

1. Kozomara A, Griffiths-Jones S: **miRBase: annotating high confidence microRNAs using deep sequencing data**. *Nucleic Acids Res* 2014, **42**(Database issue):D68-73.
2. Fromm B, Billipp T, Peck LE, Johansen M, Tarver JE, King BL, Newcomb JM, Sempere LF, Flatmark K, Hovig E *et al*: **A Uniform System for the Annotation of Vertebrate microRNA Genes and the Evolution of the Human microRNAome**. *Annual review of genetics* 2015, **49**:213-242.
3. Chan PP, Lowe TM: **GtRNAdb: a database of transfer RNA genes detected in genomic sequence**. *Nucleic Acids Res* 2009, **37**(Database issue):D93-97.
4. Langmead B, Trapnell C, Pop M, Salzberg SL: **Ultrafast and memory-efficient alignment of short DNA sequences to the human genome**. *Genome Biol* 2009, **10**(3):R25.
5. Lorenz R, Bernhart SH, Honer Zu Siederdissen C, Tafer H, Flamm C, Stadler PF, Hofacker IL: **ViennaRNA Package 2.0**. *Algorithms for molecular biology : AMB* 2011, **6**:26.
6. Li H, Handsaker B, Wysoker A, Fennell T, Ruan J, Homer N, Marth G, Abecasis G, Durbin R, Genome Project Data Processing S: **The Sequence Alignment/Map format and SAMtools**. *Bioinformatics* 2009, **25**(16):2078-2079.
7. Martin M: **Cutadapt removes adapter sequences from high-throughput sequencing reads**. *EMBnet journal* 2011, **17**.
8. Baras AS, Mitchell CJ, Myers JR, Gupta S, Weng LC, Ashton JM, Cornish TC, Pandey A, Halushka MK: **miRge - A Multiplexed Method of Processing Small RNA-Seq Data to Determine MicroRNA Entropy**. *PLoS One* 2015, **10**(11):e0143066.
9. Hafner M, Renwick N, Farazi TA, Mihailovic A, Pena JT, Tuschl T: **Barcoded cDNA library preparation for small RNA profiling by next-generation sequencing**. *Methods* 2012, **58**(2):164-170.
10. Locati MD, Terpstra I, de Leeuw WC, Kuzak M, Rauwerda H, Ensink WA, van Leeuwen S, Nehrdich U, Spaink HP, Jonker MJ *et al*: **Improving small RNA-seq by using a synthetic spike-in set for size-range quality control together with a set for data normalization**. *Nucleic Acids Res* 2015, **43**(14):e89.
11. Zhao S, Gordon W, Du S, Zhang C, He W, Xi L, Mathur S, Agostino M, Paradis T, von Schack D *et al*: **QuickMIRSeq: a pipeline for quick and accurate quantification of both known miRNAs and isomiRs by jointly processing multiple samples from microRNA sequencing**. *BMC bioinformatics* 2017, **18**(1):180.
12. Alon S, Mor E, Vigneault F, Church GM, Locatelli F, Galeano F, Gallo A, Shomron N, Eisenberg E: **Systematic identification of edited microRNAs in the human brain**. *Genome research* 2012, **22**(8):1533-1540.
13. Benjamini Y, Hochberg Y: **Controlling the false discovery rate: A practical and powerful approach to multiple testing**. *Journal of the Royal Statistical Society Series B* 1995, **57**:289.
