# Supplementary material for: miRge 2.0 for comprehensive analysis of microRNA sequencing data
Source: BMC Bioinformatics. 2018 Jul 23;19:275. doi: 10.1186/s12859-018-2287-y (PMC6112139; doi:10.1186/s12859-018-2287-y)
Supplement: Supplementary file 2 — Table S1. Features calculated for building the novel miRNA detection model. (PDF 46 kb) [file 12859_2018_2287_MOESM2_ESM.pdf]

Supplemental Table 1. Features calculated for building the novel miRNA detection model.

Feature
Sequence count in the cluster
Read count
The proportion of reads that are an exact match to the cluster sequence in the cluster
5’ Unstable length of the cluster
3’ Unstable length of the cluster
Proportion of templated nucleotide at the positions -3, -2, -1 relative to the 5' end and +1, +2, +3, +4, +5, +6 relative to the 3' end of the stable range of the cluster sequences
Proportion of non-templated adenine (A) at the positions -3, -2, -1 relative to the 5' end and +1, +2, +3, +4, +5, +6 relative to the 3' end of the stable range of the cluster sequences
Proportion of non-templated thymine (T) at the positions -3, -2, -1 relative to the 5' end and +1, +2, +3, +4, +5, +6 relative to the 3' end of the stable range of the cluster sequences
Proportion of non-templated cytosine (C) at the positions -3, -2, -1 relative to the 5' end and +1, +2, +3, +4, +5, +6 relative to the 3' end of the stable range of the cluster sequences
Proportion of non-templated guanine (G) at the positions -3, -2, -1 relative to the 5' end and +1, +2, +3, +4, +5, +6 relative to the 3' end of the stable range of the cluster sequences
The arm where the stable range of sequences were located at the precursor hairpin structure
Distance between stable range of sequences and terminal loop
Number of bindings in the stable range of sequences divided by its length
Number of hairpin loops in the precursor
Number of bindings in the precursor
Number of interior loops in the precursor
Number of nucleotides in apical loop of the precursor
Length of stem in the precursor
Minimum free energy (MFE) of the precursor
Number of bindings in the stable range of sequences
Whether UGU or UGUG motif exists in the apical loop of the precursor
Whether there is another stable range of sequences located at the other arm of the precursor
