# Supplementary material for: miRge 2.0 for comprehensive analysis of microRNA sequencing data
Source: BMC Bioinformatics. 2018 Jul 23;19:275. doi: 10.1186/s12859-018-2287-y (PMC6112139; doi:10.1186/s12859-018-2287-y)
Supplement: Supplementary file 3 — Figure S1. Distribution of non-templated nucleotides as a percentage of all 4 nucleotides at both 5′ and 3′ positions relative to the mature miRNA or equivalent non-miRNA sequence (PDF 876 kb) [file 12859_2018_2287_MOESM3_ESM.pdf]

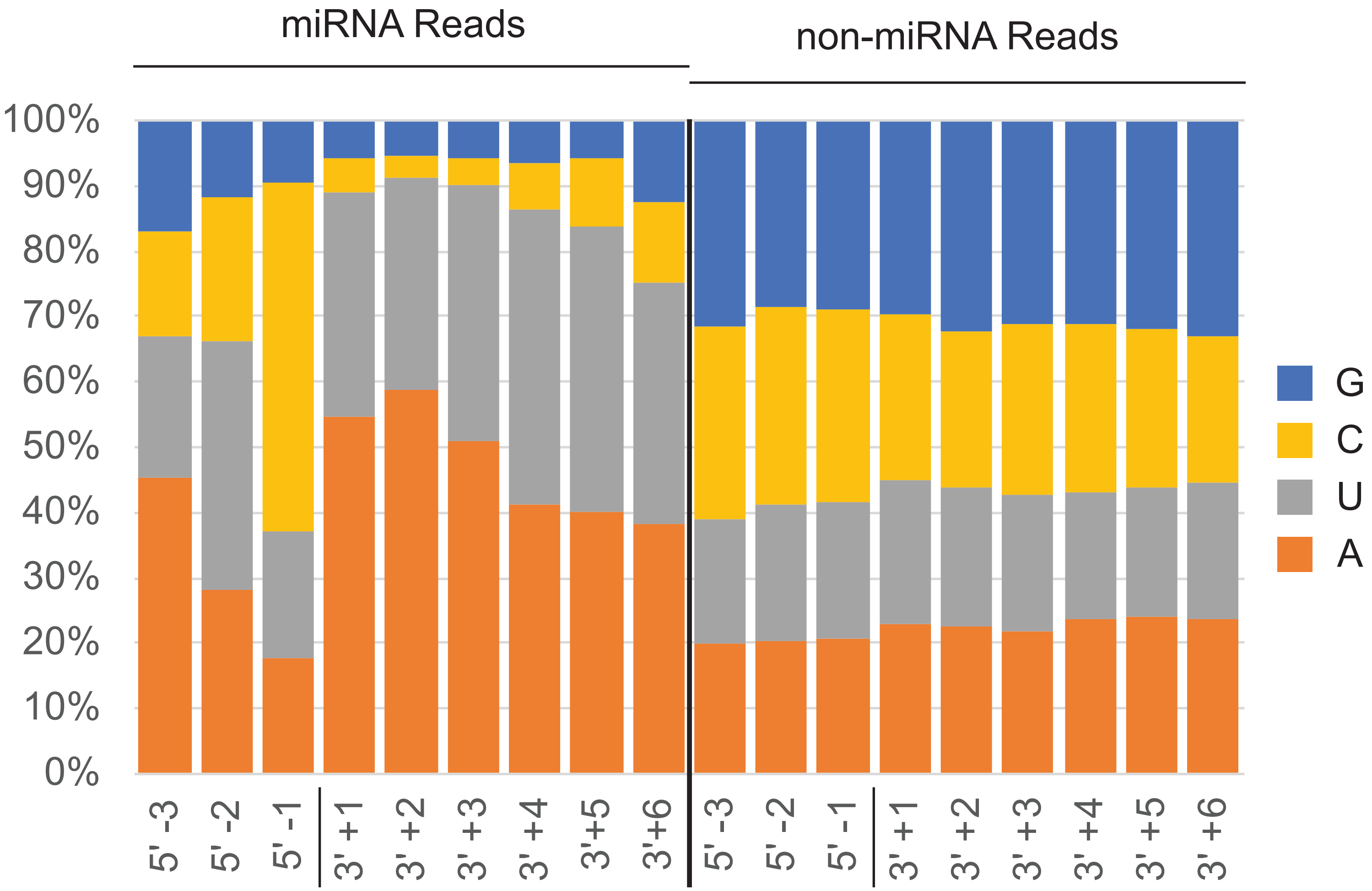

Supplemental Figure 1. Distribution of non-templated nucleotides as a percentage of all 4 nucleotides at both 5’ and 3’ positions relative to the mature miRNA or equivalent non-miRNA sequence. miRNA reads show a strong bias for non-templated A and U nucleotides in the 3’ region, consistent with our general knowledge of isomiR structure. The non-miRNA reads had consistent nucleotide usage across the reads.
