# Supplementary material for: miRge 2.0 for comprehensive analysis of microRNA sequencing data
Source: BMC Bioinformatics. 2018 Jul 23;19:275. doi: 10.1186/s12859-018-2287-y (PMC6112139; doi:10.1186/s12859-018-2287-y)
Supplement: Supplementary file 4 — Table S2. 12 rat samples for evaluating the human and mouse novel miRNA predictive models. (PDF 19 kb) [file 12859_2018_2287_MOESM4_ESM.pdf]

Supplemental Table 2. 12 rat samples for evaluating the human and mouse novel miRNA predictive models.

Tissue Type	Sequence Read Archive (SRA) References
Lung	SRR3498192, SRR3498196, SRR3498182
Liver	SRR3498150, SRR3498148, SRR3498148
Kidney	SRR3498125, SRR3498118, SRR3498130
Heart	SRR3498075, SRR3498102, SRR3498103
