# Supplementary material for: miRge 2.0 for comprehensive analysis of microRNA sequencing data
Source: BMC Bioinformatics. 2018 Jul 23;19:275. doi: 10.1186/s12859-018-2287-y (PMC6112139; doi:10.1186/s12859-018-2287-y)
Supplement: Supplementary file 5 — Table S3. All options to call in miRge 2.0. (PDF 55 kb) [file 12859_2018_2287_MOESM5_ESM.pdf]

Supplemental Table 3. All options to call in miRge 2.0.

Usage: miRge2.0 annotate [-h] [<args>]

Annotate is just to find known miRNAs and other RNA species.

optional arguments:

-h, --help	show this help message and exit
-s	[sample <required> [sample <required> ...]]<br>Two options: 1. A file where each row represents one sample name; 2. *.fastq *.fastq ...<br>or *.fastq.gz *.fastq.gz ...
-o	<dir> the directory of the outputs (default: current working directory)
-d	<string required> the miRNA database (default: miRBase. miRGeneDB is optional)
-pb	<dir required> the path to the system's bowtie binary
-lib	<dir required> the path to the miRge libraries
-sp	<string required> the species of comparison. Built in libraries are “human”, “mouse”, “fruitfly”, “nematode”, “rat” and other libraries can be built and called here<br>“zebrafish” (novel miRNA detection is confined in human and mouse)
-ps	<dir required> the path to the system's samtools binary
-pr	<dir required> the path to the system's rnafold binary
-ex	<float> the threshold of the proportion of canonical reads for the miRNAs to determine whether keeping them or not when counting. Users can set it between 0.02 and 0.5 (default: 0.1)
-ad	<string> the adapter needed to be removed, which could be illumina, ion or a defined<br>sequence (default: none)
-phred64	phred64 format (default: 64)
-spikeIn	switch to annotate spike-ins if the bowtie index files are located at the path of bowtie's<br>index files (default: off)
-tcf	switch to write trimmed and collapsed fasta file (default: off)
-di	switch to calculate isomir entropy (default: off)
-cpu	<int> the number of processors to use for trimming, qc, and alignment (default: 1)

-ai switch to calculate A to I editing (default: off)

-gff switch to output results in gff format (default: off)

--version show program's version number and exit

Usage: miRge2.0 predict [-h] [<args>]

Predict will perform annotation as above AND novel miRNA prediction.

optional arguments:

-h, --help show this help message and exit

-s [sample <required> [sample <required> ...]]

Two options: 1. A file where each row represents one sample name; 2. *.fastq *.fastq ...

or *.fastq.gz *.fastq.gz ...

-o <dir> the directory of the outputs (default: current working directory)

-d <string required> the miRNA database (default: miRBase. miRGeneDB is optional)

-pb <dir required> the path to the system's bowtie binary

-lib <dir required> the path to the miRge libraries

-sp <string required> the species of comparison. Built in libraries are “human”, “mouse”, “fruitfly”, “nematode”, “rat” and other libraries can be built and called here (novel miRNA detection is confined to human and mouse)

-ps <dir required> the path to the system's samtools binary

-pr <dir required> the path to the system's rnafold binary

-ex <float> the threshold of the proportion of canonical reads for the miRNAs to determine whether keeping them or not when counting. Users can set it between 0.02 and 0.5 (default: 0.1)

-ad <string> the adapter need to be removed which could be illumina, ion or a defined

sequence (default: none)

-phred64 phred64 format (default: 64)

-spikeIn switch to annotate spike-ins if the bowtie index files are located at the path of bowtie's

index files (default: off)

-tcf switch to write trimmed and collapsed fasta file (default: off)

-di switch to calculate isomir entropy (default: off)

-cpu	<int>	the number of processors to use for trimming, qc, and alignment (default: 1)
-ai		switch to calculate A to I editing (default: off)
-gff		switch to output results in gff format (default: off)
-ws	<file>	the file containing the overall samples to analyze for novel miRNA prediction. No header, just a list of *.fastq file names in a column. Name of file can be to your choosing (e.g. filestochecknovel.txt)
-minl	<int>	the minimum length of the retained reads for novel miRNA detection (default: 16)
-maxl	<int>	the maximum length of the retained reads for novel miRNA detection (default: 25)
-cc	<int>	the maximum read count of the retained reads for novel miRNA detection (default: 2)
-ml	<int>	the maximum number of mapping loci for the retained reads for novel miRNA detection (default: 3)
-sl	<int>	the seed length when invoking Bowtie for novel miRNA detection (default: 25)
-olc	<int>	the length of overlapped sequence when joining reads into longer sequences based on the coordinate on the genome for novel miRNA detection (default: 14)
-clc	<int>	the maximum length of the clustered sequences for novel miRNA detection (default: 30)
--version		show program's version number and exit
