# Supplementary material for: miRge 2.0 for comprehensive analysis of microRNA sequencing data
Source: BMC Bioinformatics. 2018 Jul 23;19:275. doi: 10.1186/s12859-018-2287-y (PMC6112139; doi:10.1186/s12859-018-2287-y)
Supplement: Supplementary file 6 — Table S4. Top 12 features in the mouse novel miRNA predictive model. (PDF 7 kb) [file 12859_2018_2287_MOESM6_ESM.pdf]

Supplemental Table 4. Top 12 features in the mouse novel miRNA predictive model. Hairpin structural features are labeled in bold/italics, while read compositional features are not.

Rank	Feature name	Description of the feature
1	***count_bindings_in_miRNA***	Number of bindings in the stable range of sequences
2	***pair_state_No***	Whether there is another stable range of sequences located at the other arm of precursor
3	***mFE***	Minimum free energy (MFE) of the precursor
4	***hairpin_count***	Number of hairpin loops in the precursor
5	***distanceToloop***	Distance between the stable range of sequences and the terminal loop
6	***stem_length***	Stem length of the precursor
7	exactMatchRatio	The proportion of reads that are an exact match to the cluster sequence in the cluster
8	headUnstableLength	5' unstable length of the cluster
9	***percentage_PairedInMiRNA***	Number of bindings in the stable range of sequences divided by its length
10	***pair_state_Yes***	Whether there is another stable range of sequences located at the other arm of precursor
11	***binding_count***	Number of bindings in the precursor hairpin
12	***interiorLoopCount***	Number of interior loops in the precursor
