# Supplementary material for: miRge 2.0 for comprehensive analysis of microRNA sequencing data
Source: BMC Bioinformatics. 2018 Jul 23;19:275. doi: 10.1186/s12859-018-2287-y (PMC6112139; doi:10.1186/s12859-018-2287-y)
Supplement: Supplementary file 7 — Table S5. Predictive results for 19 mouse RNA-seq datasets for novel miRNA detection. (PDF 20 kb) [file 12859_2018_2287_MOESM7_ESM.pdf]

Supplemental Table 5. Predictive results for 19 mouse RNA-seq datasets for novel miRNA detection.

Cell Type	SRA Reference	AUC	Precision	Recall	MCC
Smooth muscle cell	DRR041741	0.976	0.996	0.926	0.921
Erythroblast	DRR041759	0.976	1	0.917	0.926
Erythroblast	DRR041761	0.978	0.996	0.918	0.922
Erythroblast	DRR041762	0.977	0.996	0.916	0.929
Erythroblast	DRR041764	0.974	1	0.909	0.924
Erythroblast	DRR041765	0.976	0.991	0.909	0.922
Erythroblast	DRR041767	0.98	0.996	0.906	0.922
Erythroblast	DRR041768	0.972	0.995	0.902	0.912
Erythroblast	DRR041770	0.977	0.986	0.896	0.898
Embryo E17.5	DRR041745	0.991	0.996	0.958	0.957
Embryo E17.5	DRR041751	0.989	0.995	0.956	0.948
Embryo E12	DRR041771	0.983	0.988	0.937	0.939
Embryo E12	DRR041773	0.982	0.99	0.935	0.931
Embryo E15	DRR041774	0.984	0.992	0.926	0.93
Embryo E15	DRR041775	0.981	0.992	0.926	0.918
Embryo E18	DRR041777	0.982	0.985	0.933	0.939
Embryo E18	DRR041779	0.971	0.98	0.922	0.927
Neonate N09	DRR041780	0.982	0.995	0.947	0.951
Neonate N09	DRR041782	0.974	0.977	0.925	0.923
Mean		0.979	0.992	0.924	0.928
Std dev		0.005	0.006	0.017	0.014
